# Sclerosing angiomatoid nodular transformation presenting with thrombocytopenia after laparoscopic splenectomy - Case report and systematic review of 230 patients

**DOI:** 10.1016/j.amsu.2020.10.048

**Published:** 2020-10-29

**Authors:** Mehmet Aziret, Fahri Yılmaz, Yasin Kalpakçı, Özkan Subaşı, Adem Şentürk, Kerem Karaman, Metin Ercan

**Affiliations:** a Sakarya University Faculty of Medicine, Department of General Surgery Sakarya, Turkey; b Sakarya University Faculty of Medicine, Department of Pathology Sakarya, Turkey; c Sakarya University Faculty of Medicine, Department of Hematology Sakarya, Turkey

**Keywords:** Laparoscopy, Sclerosing angiomatoid vascular transformation (SANT), Splenectomy

## Abstract

**Background:**

Sclerosing angiomatoid vascular transformation (SANT) is a rare vascular disease of the spleen, which is difficult to diagnose due to its pre-intervention appearance of malignancy. Case Report: An 85-year-old male was transferred to our clinic for thrombocytopenia and splenic mass. A contrast enhanced abdominal CT and MRI showed nodular lesions, the largest 50mm in diameter, and several areas of heterogeneous contrast field involvement in the spleen parenchyma. Laparoscopic splenectomy was performed with normal range of platelet level. The patient's postoperative course was uneventful and he was discharged on the 6th postoperative day. Histopathology revealed SANT. The patient is now in the 18 th month of remission with platelet levels within normal range and with no recurrence.

**Results:**

Between 2004 and April 2020, a total of 230 SANT patients who underwent laparoscopic or open splenectomy or biopsy were reported in the literature. Most patients were female (52.1%), and the median age was 46 years (9 weeks-85 years). Most patients were asymptomatic (56%). Open splenectomy was performed on 166 patients (72.1%),laparoscopic splenectomy on 35 patients (15.2%) and laparoscopic partial splenectomy on 15 patients (6.5%). The median operation time and spleen weight were 143 minutes (88-213) and 260gr (68-2,720), respectively. Median follow-up time was 12 months (0-166). No recurrence was seen in patients undergoing total splenectomy.

**Conclusion:**

SANT is an unusual disease of the spleen. In the light of this systematic review, a minimally invasive method for total or partial splenectomy,specifically laparoscopy, can be preferred as the treatment of choice.

## Highlights

• SANT is a rare, benign vascular disease of the spleen.
• The correct diagnosis of SANT pre-intervention or preoperatively is a significant for treatment.
• Laparoscopic splenectomy is curative treatment for SANT.

## 1 Introduction

Sclerosing angiomatoid nodular transformation (SANT) is a rare benign vascular disease of the spleen [1,2]. The first cases of SANT were recorded by Martel et al., after the pathological examination of 25 patients [2]. But it has been reported that SANT can be associated with Epstein-Barr virus (EBV) infection and immunoglobulin (Ig)G4-related sclerosing disease [5,8]. Patients with SANT are commonly asymptomatic, but some abdominal pain or discomfort, fullness, distention, left upper quadrant pain, flank pain, fever or night sweats, weight loss, dyspepsia, epigastralgia may be seen in certain patients [[7], [8], [9]]. Despite the increased sensitivity, quality and accuracy of imaging methods such as contrast-enhanced computed tomography and magnetic resonance that technological developments have brought, an absolute diagnosis of SANT cannot be made without immunohistochemical examination [[10], [11], [12]]. For this reason, prior to intervention or operation, an incorrect diagnosis of malignity is frequently assumed. In fact, hemangioma, hamartoma, inflammatory pseudotumor, lymphoma, angiosarcoma, littoral angioma and metastasis should be considered primarily in differential diagnosis [[13], [14], [15], [16], [17]].

With this study, we aim to report the successful management of a SANT patient after their laparoscopic splenectomy (LS) and introduce a systematic review of the literature on this topic.

## 2 Material and methods

### 2.1 Research protocol, strategies and selection criteria

The identification and data extraction for the present study were carried out by searching Google Scholar, PubMed, and Research-gate databases using the following search terms: ‘spleen’, ‘splenectomy’, laparoscopic splenectomy’, ‘laparoscopic partial splenectomy’, ‘sclerosing angiomatoid nodular transformation, ‘SANT’ and ‘biopsy’. Moreover, informed researchers manually examined other appropriate references for additional relevant studies. The article's title, abstract and full text were evaluated according to inclusion and exclusion criteria. To avoid misinformation, studies in languages other than English or without full-text availability were not included. This systematic literature review comprised all articles from 2004 to April 2020. The following information was gathered: first author names; age, sex and body mass index (BMI) of patients; symptoms, co-morbidities, clinical features, level of tumor markers, and splenomegaly; pre-intervention or referring diagnosis; type of splenectomy (laparoscopic, open or partial splenectomy), operation time and length of hospital stay; spleen weight, diameter of nodule(s), fibrous or stromal scaring, and multinodular state; complications, follow-up and outcome.

**Fig. 1:**
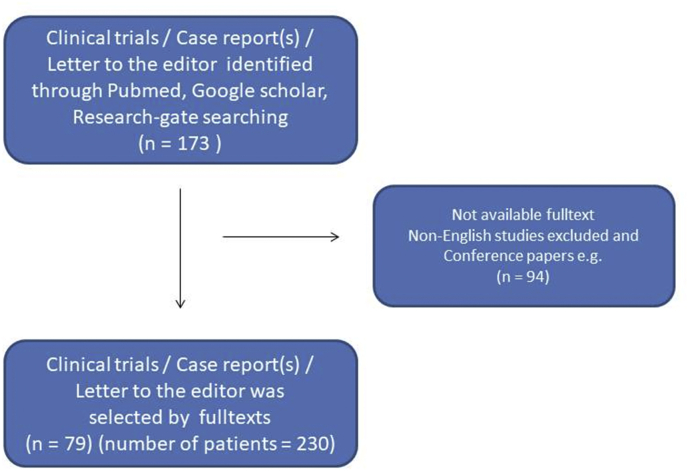
Flow chart of the study.

Finally, a research flow-chart was created to include all data evaluation (Fig. 1). The present study was edited by SCARE 2018 guidelines [82].

## 3 Results

### 3.1 Case report

An 85-year-old man with splenic mass was admitted to our clinic. Fifteen days previously, the patient had been hospitalized in the hematology clinic for thrombocytopenia, with complaints of tongue bruising. At 170 cm tall and weighing 68 kg (BMI: 22.4 kg/m^2^), his anamnesis revealed the use of Prednisolone (80 mg, perioral, daily) for thrombocytopenia. His history included a prostate operation for benign prostate hyperplasia, appendectomy and inguinal hernia repair. The anamnesis also revealed no co-morbidities such as diabetes mellitus (DM), hypertension (HT) or coagulopathy disorders, and he was a non-smoker. Furthermore, the patient didn't have drug history, including any relevant genetic information, and psychosocial history. The spleen was not palpable on physical exam. Following prednisolone treatment, the white blood cell level was 9.300/mm^3^, international normalized ratio (INR) was 1.02 (normal range: 0.8–1.29) and platelet levels were 437 K/Ul (normal range:142–427). Moreover, levels of CEA (Carcinoembriogenic Antigen) and CA 19–9 (Carbohydrate Antigen) were 4.3 ng/ml (normal range: 0–5) and 25.5 U/ml (normal range 0–37), respectively.

**Fig. 2:**
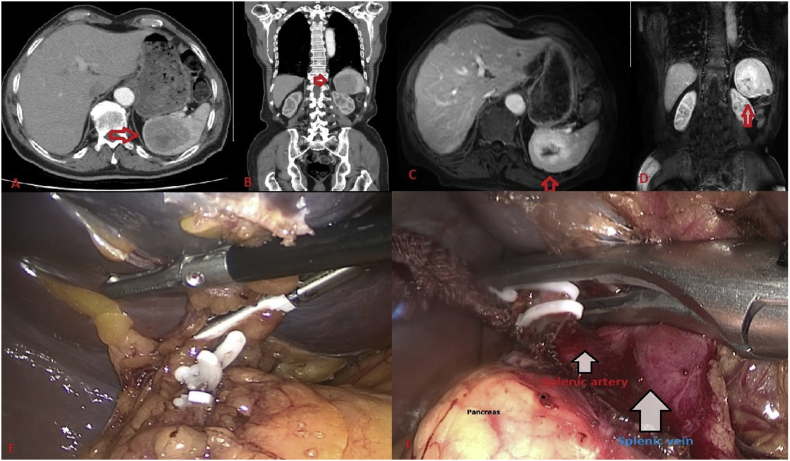
Abdominal CT (A&B) and Abdominal MR (C&D) show SANT with red arrows. (For interpretation of the references to color in this figure legend, the reader is referred to the Web version of this article.)

Abdominal ultrasound revealed a solid lesion that was hypoechoic, exophytic and 80mm in diameter, located in the mid-superior spleen. A contrast enhanced abdominal CT showed several nodular lesions, the largest of which was 50 mm in diameter, and several heterogeneous contrast field involvements with hypodense expansive character in the spleen parenchyma, considered to be possibly metastasis or abscess (Fig. 2A). A contrast-enhanced abdominal magnetic resonance imaging revealed a splenic mass of 67 mm in diameter, with central cystic necrosis and peripheral contrast field involvement anteromedial to the spleen (Fig. 2B). The patient was transferred from the hematology clinic to our clinic for a splenectomy with diagnosis of spleen mass and thrombocytopenia. After oncology consultation, we presumed a malignity or metastasis in the spleen, and splenectomy was planned. Addison's prednisolone protocol was used prior to the operation. Laparoscopic splenectomy was performed by senior surgeon after anesthesia consultation gave an ASA score of III (American Society of Anesthesiologists).

**Fig. 3:**
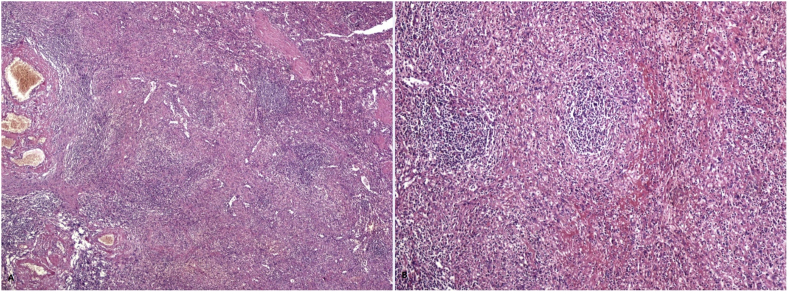
Dilated vessel sections and nodular structures with spleen tissue (A) (Hematoxylin & Eosin X 100) and small nodules structures around the connective tissue (B) (Hematoxylin & Eosin X200).

A three-port technique and North American method was used for the laparoscopic splenectomy. A 10-mm port was inserted under the arcus costarum in the midclavicular line using a Veress needle. Pneumoperitoneum was performed with carbon dioxide (CO2), and the intra-abdominal pressure was maintained at 12 mmHg. Next, a 5-mm trocar was inserted into the epigastric area just inferior to the xiphoid process. A 10-mm trocar was inserted into the left upper quadrant for direct 30°-telescope vision (Fig. 3). Indirect splenic hilum dissection method was used, as described by Aziret et al. [18]. A 5-mm bipolar vessel sealer was used for dissection and ligation of the spleen (splenocolic, splenophrenic and splenogastric). Once dissection of the base of the spleen was performed, the splenic hilum was revealed. The splenic vein and splenic artery were ligated using vascular clips under direct vision. The splenectomy specimen was placed in a large retrieval bag and removed through the left upper quadrant by slightly extending this incision. Following drainage of the splenectomy area, the operation was completed without any complication. The patient's postoperative course was uneventful, and he tolerated successfully the postoperative period without complication. The platelet level increased to within the normal range, and he was discharged on the 6th postoperative day with the decision to reduce the prednisolone dosage. No infection Epstein-Barr virus (EBV) infection or IGg4 were detected.

**Fig. 4:**
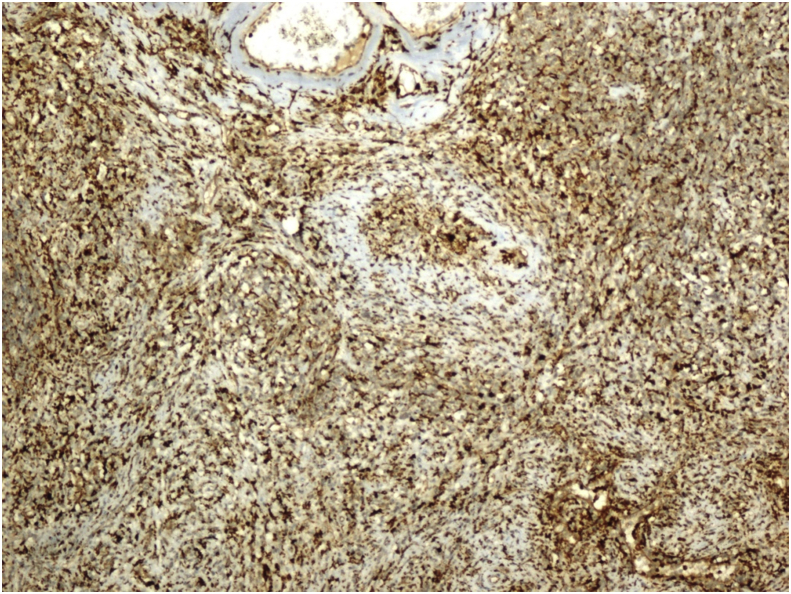
Diffuse CD68 positivity on immunohistochemical staining (X200).

**Fig. 5:**
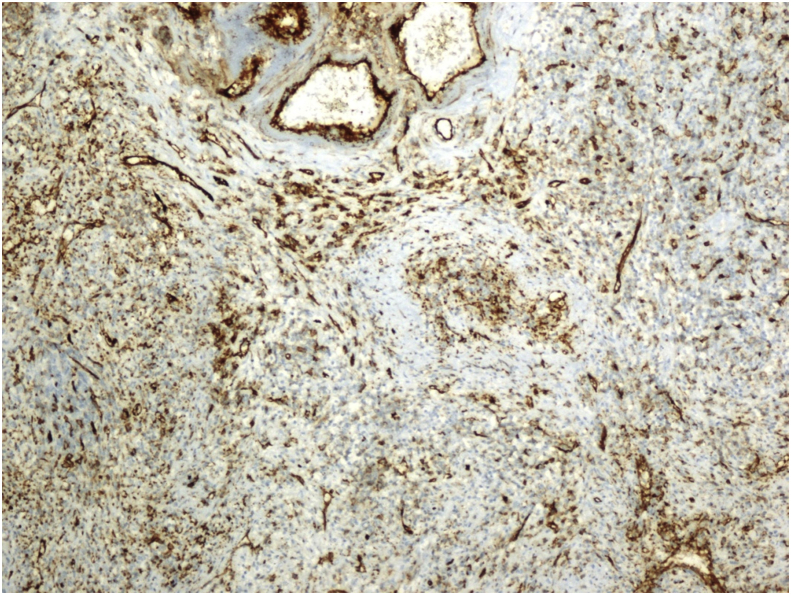
CD31 positive capillaries and small veins (Immunohistochemical staining) (X200).

**Fig. 6:**
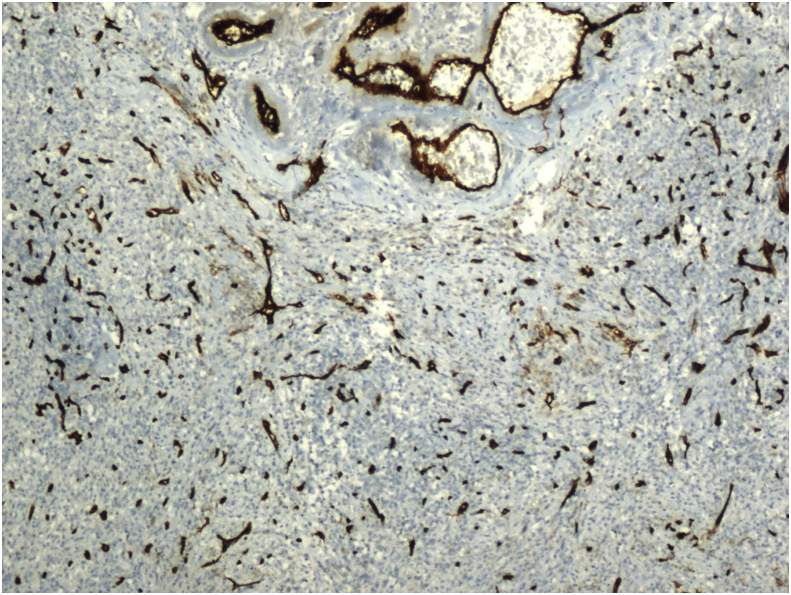
CD34 positive capillaries (Immunohistochemical staining) (X200).

Histopathological examination revealed the sclerosing angiomatoid nodular transformation (SANT). Macroscopic examination revealed a lesion with irregular margins 6 × 4.5 × 4 cm, and spleen material weighing 179 g and 9 × 8x4.5 cm in size. Under microscopic examination, the splenic mass was seen to be composed of multiple nodules of numerous vascular sections, consisting of spindle and oval round cells surrounded by hyalinized connective tissue (Fig. 3). Immunohistochemical staining of macrophages within the lesion were positive for CD68 (Fig. 4). In addition, capillaries and small veins were positive for CD31, and capillaries were also positive for CD34 (Fig. 5, Fig. 6). The patient is now 18 months postoperative with a platelet level of normal range and with no recurrence. Moreover, the patient reported the perspective on the treatments was affirmative in postoperative period and follow-up.

## 4 Systematic literature review

### 4.1 Patients and characteristics

A total of 230 SANT patients undergoing laparoscopic, open splenectomy or biopsy between 2004 and April 2020 were reported on in English language literature [[6], [7], [8], [9], [10], [11], [12], [13], [14], [15], [16], [17], [18], [19], [20], [21], [22], [23], [24], [25], [26], [27], [28], [29], [30], [31], [32], [33], [34], [35], [36], [37], [38], [39], [40], [41], [42], [43], [44], [45], [46]]. Patient information was only retrieved from English language medical databases with full-texts, using Google Scholar, PubMed, Scopus and Research-gate. These English language medical databases provided 68 case reports, a letter to the editor, a clinical vignette and 10 retrospective cohort studies. Most of the patients were female (52.1%; 120 cases). The median age was 46 years (min-max: 9 weeks–85 years) and the median BMI was 21.7 kg/m^2^ (min-max: 17-23-74.6). Most comorbidities were malignities unrelated to the spleen (16%; n = 37), anemia (6.9%; n = 16), and benign liver or gallbladder disease (4.7%; n = 11). The most common symptoms were abdominal pain (6.9%), abdominal discomfort, mass, fullness, distention (6.9%) and left upper (quadrant) pain, or flank pain (6%). However, most patients were asymptomatic (56%).

### 4.2 Clinical features, clinical diagnosis and surgery

Splenomegaly was seen in 19 patients (8.2%). In patients with this condition the rate for normal range tumor marker levels was 22.1%, compared to 77.3%. For those without. The pre-intervention or clinical diagnosis was SANT in 26.9% of cases, malignancy or suspected malignancy in 23%, splenic mass or splenic (nodular) lesion in 15.6%, hemangioma or hemangioendothelioma in 12.1%, IPT in 8.6% and tumor metastasis in 6.5%. Splenectomy was performed on a total of 166 patients (72.1%): laparoscopic splenectomy on 35 patients (15.2%), laparoscopic partial splenectomy on 15 patients (6.5%), laparotomy and splenectomy ± gastrectomy on 4 patients (1.7%), partial splenectomy on 3 patients (1.3%) and hand-assisted laparoscopic splenectomy on 2 patients (0.8%). Biopsy (+/− splenectomy) was performed on 8 patients (3.4%) but a definite diagnosis of SANT could be made in only 3 cases (1.3%).

### 4.3 Management and outcomes of SANT

The median operation time was 143 minutes (min-max: 88–213). The median discharge time after splenectomy was 5.5 days (min-max: 2–14). The median spleen weight was 260gr (min-max:68–2720). The average diameter of nodules was 5.2cm (min-max: 1.2–17). Most patients (>95%) with SANT had fibrous scars or stroma, and multinodular status. The median follow-up was 12 months (min-max: 0–166). No recurrence was detected in any patient receiving total splenectomy. Of patients who had undergone biopsy without having splenectomy, only 2 had recurrence. Complications mentioned in the literature include suture granuloma in one patient (0.4%), fluid collection followed by drainage in one patient (0.4%), atrial fibrillation in one patient (0.4%), portosplenic vein thrombosis in one patient (0.4%), and multiorgan failure in one patient (0.4%). Moreover, complications of Grade II and Grade IIIa according to the Clavien-Dindo scale were seen in 2 patients in the open splenectomy group, 2 in the laparoscopic splenectomy group and in no patients undergoing laparoscopic partial splenectomy.

## 5 Discussion

This is the first systematic review of 230 patients mentioned in the literature. After careful evaluation of 230 patients, we noticed that the occurrence of SANT is slightly higher in female patients than in males (52.1% female; 47.9% male). The median age in this study is lower compared to previous reviews, at 46 years (min-max: 9 weeks–85 years). We believe this decrease may stem from improved access to more effective imaging methods. Martel [2], who first identified and used the term SANT, reported a series of 25 cases, 17 female and 8 male, with a median age of 56 years (8 weeks - 85 years). Martel [2] described SANT as a solitary lesion, well-circumscribed, with a multi-nodular angiomatoid appearance, and a peculiar heterogeneous immunostaining profile for vascular markers.

In the literature, certain comorbidities were reported in SANT patients; especially, malignity unrelated to spleen disease (16%), anemia (6.9%), benign liver or gallbladder disease (4.7%) and hypertension (4.3%), [[1], [2], [3], [4], [5], [6], [7], [8], [9], [10], [11], [12], [13], [14]]. Moreover, SANT may be indirectly associated with other cases of malignity that have comorbidities (16%, n = 37) (colorectal cancer 2.1%, gastric cancer 2.1% and lung cancer 1.3%) as SANT was detected incidentally during the follow-up of primary malignities [[5], [6], [7], [8], [9], [10], [11], [12], [13], [14], [15], [16], [17]]. The literature reports three cases of thrombocytopenia in patients with SANT [6,73,77]. Dieobold et al. [6] reported a patient presenting with thrombocytopenia due to paramyxovirus 19. Gooch et al. [73] noted that the surgical oncology consultation agreed that a splenic mass was the most likely cause of thrombocytopenia in their patient. Finally, Pelizzo et al. [77] reported a case of SANT occurring in a nine-week-old female infant who was admitted with severe abdominal distension and rectal bleeding, severe anemia (Hb 4.6 g/dL), thrombocytopenia (54 × 103/μL) and coagulation abnormalities resulting from persistent abdominal hemorrhaging from ruptures of the spleen capsule that were un-responsive to blood transfusions. In the present study, no infection such as Epstein-Barr virus (EBV) infection or IGg4 was detected, and we presumed SANT to be the cause of the thrombocytopenia as the patient had normal range platelet levels after laparoscopic splenectomy.

Most SANT patients are asymptomatic; however, when symptoms present in clinical practice, they cover a wide spectrum, and are non-specific in nature; they include abdominal pain, abdominal discomfort, abdominal fullness or distention and left upper (quadrant) pain, flank pain, fever or night sweats, weight loss, dyspepsia, epigastric pain, nausea or vomiting, fatigue and joint pain [17,19,20]. As patients are generally asymptomatic, or have no tangible symptoms, SANT is generally detected incidentally during imaging [[19], [20], [21], [22], [23], [24], [25]].

Splenomegaly can occur in benign or malign diseases of the spleen. As spleen volume extends, the spleen can be palpated during physical examination or imaging. Although clear information about splenomegaly is not available in the literature for all cases, those spleen weights given were mostly normal (19 patients; 8.2%) [2,6,[15], [16], [17],^26^,^37^,^57^,^61^,^77^].

The correct diagnosis of SANT pre-intervention or preoperatively is a significant problem in clinical practice because in imaging it presents with the same features as a malignancy. Therefore radiology-pathology corroboration is highly recommended [[21], [22], [23], [24], [25], [26], [27], [28], [29], [30]]. A multidisciplinary approach should include specialists in radiology, gastrointestinal surgery, pathology and gastrointestinal oncology in order to evaluate the possibility of malignity in SANT patients [[31], [32], [33],^35^,^36^]. Lewis et al. [34] showed that SANT is usually detectable in typical MRI and CT findings, such as its following portal venous or arterial phase peripheral enhancing radiating lines, progressive enhancement, and T2-Hypointense signal intensity. Moreover, peripheral enhancing lines and, in some patients, rim enhancement correspond histologically to multiple angiomatous nodules concentrated around the periphery and in a radiating, spoke wheel pattern [34]. In this study, a definite diagnosis of SANT was made in 62 patients (26.9%), pre-intervention or preoperatively, using radiology imaging. Of the 230 patients, a splenic biopsy (trucut or fine-needle biopsy) was performed to establish diagnosis in 8 patients, but an accurate diagnosis was made in only 3 cases, while the remaining patients underwent splenectomy. Possible complications after spleen biopsy include hemorrhage and injuries to the small intestine, colon, diaphragm and lung [5,13,23,31,47,54,58,69].

The weight of splenectomy specimens in the literature varies from 68 to 2720 g, most cases being recorded after total splenectomy. During operation or macroscopic examination, certain signs may be visible; particularly, the mass appears as multiple individual and confluent nodules with sizes ranging from 1.2 to 17 cm (median size:5.2 cm), well-circumscribed, non-encapsulated, a bosselated mass with multiple dark brown nodules (bleed areas in angiomatoid nodules) interspersed with fibrotic stroma [24,[37], [38], [39], [40]]. In the literature, most authors (>95%) reported multiple nodules and fibrotic stroma or scars [2,20,[40], [41], [42], [43], [44], [45]]. In microscopic examination, all the cases have shown a spectacularly stable appearance at low magnification, best characterized as multiple angiomatous nodules in a fibro-sclerotic stroma. Also, the individual nodules are generally round but occasionally convoluted, of variable size, widely separated or coalescent [2]. Furthermore, in immunohistochemical studies, Martel et al. [2] noted 3 different types of blood vessels within the angiomatoid nodules. Firstly, CD34 highlighted narrow, well-formed capillaries in a well-organized, near-lobular pattern; these capillaries were CD8-negative and CD31-positive. Secondly, some but not all nodules displayed a few open vascular channels that were moderately or weakly CD8 positive, consistent with splenic sinusoids; these vessels were CD34-negative and CD31-positive. Thirdly, CD31 highlighted numerous cells within the angiomatous nodules, counting many single cells and all recognizable vascular channels, resulting in unusual complex meshwork [2].

The curative treatment for SANT is splenectomy [46,[48], [49], [50], [51], [52], [53],[55], [56], [57], [58], [59], [60]]. Open splenectomy or minimally invasive splenectomy are both options for surgical treatment [[62], [63], [64], [65], [66], [67], [68], [70], [71], [72], [73], [74], [75], [76], [77]]. In recent years, minimally invasive surgery such as robotic or laparoscopic splenectomy has become more popular due to a shorter hospital stay, less postoperative abdominal pain, and an early return to normal activity [18,[63], [64], [65],^78^]. After total splenectomy, no evidence of recurrence was reported in the follow-up period (median follow-up:12 months (0–166)).

The spleen performs a crucial role with regard to erythrocytes and the immune system.

The spleen synthesizes antibodies and removes antibody-coated bacteria and blood cells through blood and lymph node circulation. The spleen is the main center of movement of the mononuclear phagocyte system (MPS) and is like a large lymph node; asplenia or spleen removal can trigger a tendency to important infections. For this reason, Budzyński and other authors have recommended laparoscopic partial splenectomy [6,79,81]. Following splenectomy, complications including suture granuloma, fluid collection, atrial fibrillation, portosplenic vein thrombosis and multi-organ failure can occur; care must be taken during the perioperative period to avoid morbidity or mortality [2,5,65,71,72,80,81].

There are two main limitations to the present study. First, the patient's data was not available, for instance; body mass index, operation time, length of the hospital stay e.g. Secondly, the number of the clinical trials was lower than case report (s) (series) or letter to the editor in literature.

## 6 Conclusion

SANT is a rare, benign vascular disease of the spleen. Accurate diagnosis of SANT preoperatively can be a challenge and requires radiology-pathology corroboration. In the light of this systematic review, we consider that total or partial splenectomy should be carried out using minimally invasive treatment methods, preferably laparoscopy, to achieve the best results.

## Learning Points

• Sclerosing angiomatoid nodular transformation (SANT) is benign vascular disease of the spleen which can mimic the malign features in imaging methods.
• The diagnosis of SANT requires clearly assessed the computed tomography, magnetic resonance imaging and pathological examination of the spleen mass.
• Laparoscopic splenectomy is usually preferred for treatment of SANT.
• Recently, laparoscopic partial splenectomy has become to perform in some cases for SANT.

## Provenance and peer review

Not commissioned, externally peer-reviewed.

## Funding

No funding.

## Declaration of competing interest

There is no conflict of interest.
